# 1α, 25-Dihydroxyvitamin D3 protects gastric mucosa epithelial cells against *Helicobacter pylori*-infected apoptosis through a vitamin D receptor-dependent c-Raf/MEK/ERK pathway

**DOI:** 10.1080/13880209.2022.2058559

**Published:** 2022-05-19

**Authors:** Shuai Zhao, Daihong Wan, Yaoyao Zhong, Xiwei Xu

**Affiliations:** a Department of Gastroenterology, Beijing Children’s Hospital, Capital Medical University, National Center for Children’s Health, Beijing, China; b Pediatric Department of Yantai Yuhuangding Hospital, Qingdao University, Yantai, China; c Pediatric Department of Qingdao Women and Children’s Hospital, Qingdao University, Qingdao, China

**Keywords:** Cell proliferation, Bcl-2 family, caspase

## Abstract

**Context:**

Due to the resistance of *Helicobacter pylori* to antibiotics, it is difficult to eradicate this pathogenic bacterium from the host. The role of 1α, 25-dihydroxyvitamin D3 (1,25-D3) in *H. pylori*-infected gastric mucosa epithelial cells remains unknown.

**Objective:**

This study investigates the protective property of 1,25-D3 against *H. pylori*-infected apoptosis in gastric mucosa epithelial cells and its potential molecular mechanisms.

**Materials and methods:**

GES-1 cells were infected with *H. pylori* SS1 strain (MOI: 100) and treated with 1,25-D3 at 100, 200, and 300 nM for 24 h. Mice were orally gavaged with 10^8^ CFUs of *H. pylori* and 25 µg/kg 1,25-D3 every other day for 1 month. CCK-8, LDH assay, TUNEL assay and western blot were used to determine the effect of 1,25-D3 on *H. pylori*-induced apoptosis.

**Results:**

*H. pylori* infection decreased cell viability to 59.2%, while 100–300 nM 1,25-D3 increased cell viability to 62.2%, 78.4% and 87.1%, respectively. Compared with positive control (4.53-fold), 1,25-D3 reduced caspase-3 activity to 4.49-, 2.88- and 1.49-fold, reduced caspase-6 activity to 2.36-, 1.88- and 1.50-fold, reduced caspase-9 activity to 4.55-, 2.91- and 2.01-fold. 1,25-D3 alters Bcl-2 family, caspase protein expression and c-Raf/MEK/ERK phosphorylation levels *in vivo* and *in vitro*. Suppression of 1,25-D3 in apoptosis was reliant on binding to vitamin D receptor. The pharmacological inhibition of c-Raf/MEK/ERK phosphorylation blocked the anti-apoptotic effect of 1,25-D3.

**Discussion and conclusion:**

1,25-D3 protected gastric mucosa epithelial cells against *H. pylori*-infected apoptosis through a VDR-dependent c-Raf/MEK/ERK pathway.

## Introduction

*Helicobacter pylori* is a common human Gram-negative bacillus, which is perhaps the most infectious of all known pathogenic bacterium (Camilo et al. 2017). However, it is difficult to eradicate this pathogenic bacterium using immune cells from the host (Gu 2017). *H. pylori* is a flagellated microaerophilic bacillus and colonises the human gastrointestinal mucosa, which leads to gastritis, peptic ulcers, mucosa-associated lymphoid tumours and gastric adenocarcinoma (Burucoa and Axon 2017). According to statistics, almost half of the world’s population have been infected with varying prevalence in different countries (Cover and Blaser 2009). A standard quadruple therapy consisting of a proton pump inhibitors (omeprazole), two antibiotics (amoxicillin/clarithromycin) and bismuth pectin is widely used as the first-line strategy for infection treatment. However, the resistance of *H. pylori* to clarithromycin and metronidazole significantly increases, which results in the eradication rate decreasing by this therapy (O'Connor et al. 2019).

Vitamin D, as an important component of the vitamin D hormone system, is synthesised in the skin under ultraviolet irradiation. 7-Dehydrocholesterol (7-DHC) is catalysed into cholecalciferol (vitamin D3, VD3). VD3 is subsequently hydroxylated to form 25-hydroxyvitamin D3 in the liver and then to yield the biologically active form of vitamin D3 (1α, 25-dihydroxyVD3, 1,25-D3) in the kidney (Cantorna et al. 2015). One of the pathogenic mechanisms of infection is that secreted enzymes and cytotoxic proteins from *H. pylori* damage gastric epithelial cells and induce apoptosis (Xia and Talley 2001). Both the caspase-9-mediated mitochondrial pathway and the caspase-8-mediated extrinsic pathway were involved in *H. pylori*-induced apoptosis (Chattopadhyay et al. 2010). Vitamin D receptor (VDR) activated by 1,25-D3 exhibits various physiological functions including calcium and phosphate homeostasis, bone metabolism, immunomodulation, cell growth and differentiation and transcriptional regulation in many tissues (Valdivielso and Fernandez 2006). Following VDR ligand stimulation such as 1,25-D3, VDR is translocated from cytoplasm to nucleus, and then forms a heterodimer with retinoid X receptor (RXR) to regulate gene transcription (Deuster et al. 2017). It has been reported that VD3 activates the autolysosomal degradation function against *H. pylori* in gastric mucosa epithelial cells through the protein disulphide isomerase family A member 3 (PDIA3) receptor, which is a non-classical VDR (Hu et al. 2019). However, the protective effect of 1,25-D3 on *H. pylori*-induced apoptosis in gastric mucosa epithelial cells remains unknown.

In the present study, we sought to delineate the protective effect of 1,25-D3 and its underlying mechanism on *H. pylori*-infected gastric mucosa epithelial cells. We demonstrated that 1,25-D3 provided a protective effect against *H. pylori*-induced apoptosis through activation of the c-Raf/MEK/ERK pathway, and provides a synergistic effect for the standard quadruple therapy in the eradication of *H. pylori*.

## Materials and methods

### Reagents, antibodies and commercial kits

1,25-D3 was purchased from Sigma Chemical Co.(St. Louis, MO, USA). Primary antibodies including anti-Bcl-2 (#4223), anti-Bax (#5023), anti-Bcl-xL (#2764), anti-Bak (#12105), anti-caspase-3 (#14220), anti-caspase-6 (#9762), anti-caspase-9 (#9502), anti-VDAC (#4866), anti-AIF (#5318), cytochrome c (#11940), anti-c-Raf (#53745), anti-phospho-c-Raf (#9427), anti-MEK1/2 (#4694), anti-phospho-MEK1/2 (#9154), anti-ERK1/2 (#4695), anti-phospho-ERK1/2 (#4370), anti-RXRα (#3085), anti-VDR (#12550), anti-GAPDH (#97166) and secondary anti-mouse or anti-rabbit antibodies were purchased from Cell Signalling Technology (Danvers, MA, USA). LDH and CCK-8 commercial kits were purchased from Jiancheng Institute of Biotechnology (Nanjing, China). Caspase-3, −6 and −9 activity kits were purchased from Beyotime Institute of Biotechnology (Nantong, China). Terminal deoxynucleotidyl transferase (TdT)-mediated dUTP-digoxigenin nick-end labelling (TUNEL) kit was purchased from Ribobio Biotechnology Co., Ltd. (Guangzhou, China). MEK1/2 inhibitor U0126 was purchased from Cell Signalling Technology. Lipofectamine™ RNAiMAX transfection reagent was purchased from Thermo Fisher Scientific (Waltham, MA, USA). RIPA buffer (#9806), protease inhibitor cocktail (#7012) and phosphatase inhibitor cocktail (#5870) were purchased from Cell Signalling Technology.

### Cell and bacterial culture

Human gastric mucosa epithelial cell line GES-1 was purchased from KeyGen Biotech (Nanjing, China) and cultured in Dulbecco’s modified Eagle’s medium (Thermo Fisher Scientific, Waltham, MA, USA) supplemented with 10% foetal bovine serum (Gibco, Grand Island, NY, USA) at 37 °C in a humidified atmosphere of 5% CO_2_. The *H. pylori* Sydney strain 1 (SS1) was routinely grown on tryptic soy agar (BD #236950) plates supplemented with 5% sheep blood in mixed air containing 10% CO_2_, 5% O_2_, and 8% N_2_ at 37 °C.

### *H. pylori* infection animal model and treatment

C57BL/6 male mice (6-8 weeks age) were obtained from Charles river (Beijing, China). All mice were housed in plastic cages under a 12 h light/dark cycle with free access to water and food. The animal procedure was performed with the approval of the Laboratory Animals Ethics Committee of Capital Medical University. Mice were orally gavaged with 10^8^ CFUs of *H. pylori* and 25 µg/kg 1,25-D3 every other day for 1 month (two groups, *n* = 8). After anaesthesia by a intraperitoneal injection of ketamine (100 mg/kg) and xylazine (5 mg/kg), mice were sacrificed and stomach tissues were collected for western blot analysis.

### siRNA transient transfection

The siRNAs for VDR (#s14777) and scramble siRNA (#4390843) were synthesised by Life Technology. Briefly, GES-1 cells were cultured in 6-well plate and transfected with the 100 nM siRNA for 12 h by Lipofectamine™ RNAiMAX transfection reagent according to the manufacturer’s instructions.

### CCK-8, LDH and caspase activity assay

The effects of the 1,25-D3 on the viability of the human gastric mucosa epithelial cell line GES-1 were evaluated by CCK-8 and LDH assay. GES-1 cells (1 × 10^4^ per well) were seeded into 96-well plates for 24 h and treated with different concentrations of 1,25-D3 in the absence or presence of *H. pylori* for 24 h at 37 °C, CCK-8, LDH and Caspase activity assay were performed according to the manufacturer’s instructions.

### TUNEL assay

The TUNEL assay was performed according to the manufacturer’s instructions. Briefly, GES-1 cells were plated in a 6-well plate and treated with *H. pylori* and 200 nM 1,25-D3 for 24 h. Then, cells were fixed with 4% paraformaldehyde for 10 min, washed with PBS twice and treated with 20 μg/mL proteinase K at 37 °C for 20 min. The cells were washed with PBS twice and stained with DAPI staining solution for 10 min. The stained cells were washed with PBS twice and visualised with a fluorescence microscope (magnification, 200×; Nikon Corporation, Tokyo, Japan).

### Western blot analysis

Cells plated in a 6-well plate were lysed in radio immunoprecipitation assay (RIPA) lysis buffer containing protease inhibitor cocktail and phosphatase inhibitor cocktail for 30 min on ice. Then cells were harvested and centrifuged for 10 min at 13,000 *g* at 4 °C. The supernatant was collected and the concentration of protein sample was determined by BCA protein assay kit (Beyotime Institute of Biotechnology, Nantong, China). For stomach protein extraction, tissue was homogenized in ice-cold RIPA buffer, centrifugated at 12,000 *g* and supernatant was harvested. The sample (40 µg) was loaded to each well, separated by SDS-PAGE, electroblotted onto PVDF membranes and blocked in 5% blocking buffer (5% non-fat dry milk) for 1 h. The membranes were incubated with indicated antibodies overnight at 4 °C and then incubated for 1 h with secondary antibody tagged with horseradish peroxidase at room temperature. Signals were visualised LumiGLO reagent® (Cell Signalling Technology, #7003). GAPDH was used as loading control. The densitometry analysis was performed by Image J software (NIH, USA).

### Statistical analysis

Statistical analysis was performed using unpaired Student’s *t*-test or ANOVA (GraphPad Prisim, CA). All data are expressed as mean ± standard error of mean (SEM). Differences were considered statistically significant at *P* < 0.05.

## Results

### 1,25-D3 promotes cell proliferation in *H. pylori-*infected GES-1 cells

**Figure 1.**
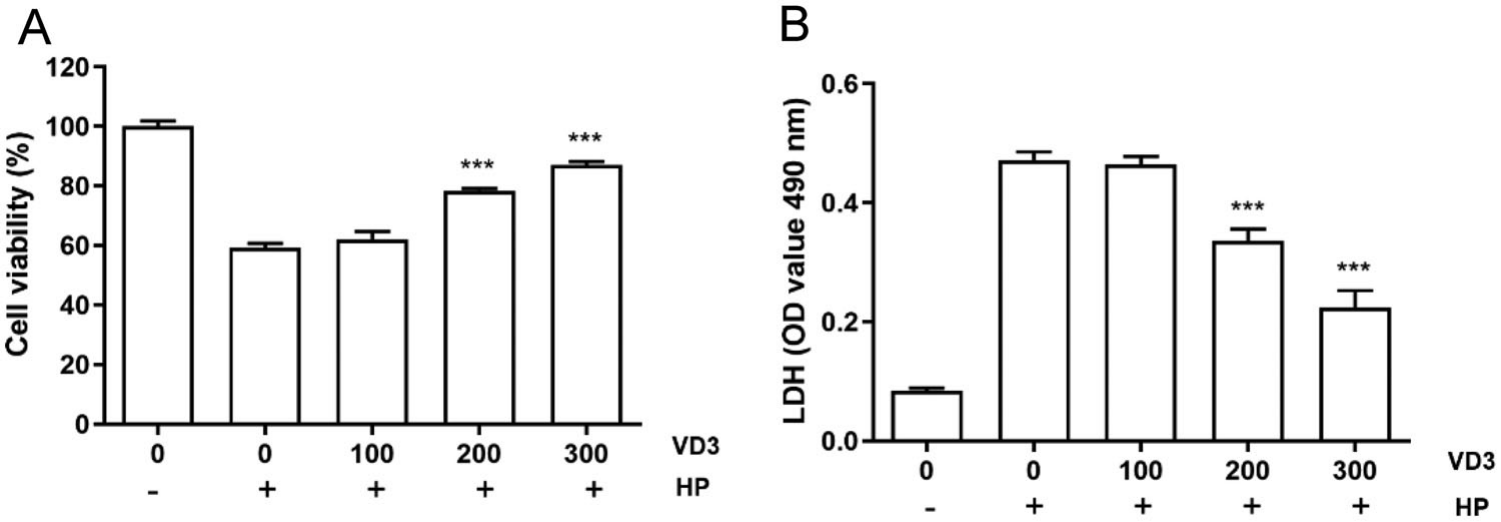
1,25-D3 promotes cell proliferation in *H. pylori*-infected GES-1 cells. (A) GES-1 cells were infected with *H. pylori* SS1 strain (MOI: 100) and treated with differernt concetrations of 1,25-D3 for 24 h, the cell viability was determined by CCK-8 assay. (B) GES-1 cells were infected with *H. pylori* SS1 strain (MOI: 100) and treated with different concentrations of 1,25-D3 for 24 h, LDH releasing was determined by a commercially available LDH assay kit. Bars represent means ± S.E.M of three independent experiments. ***p < 0.001 vs. *H. pylori* alone treatment.

We attempted to determine the effects of 1,25-D3 on cell proliferation in *H. pylori*-infected GES-1cells. CCK-8 assay results showed that *H. pylori* caused a significant decreasing of cell proliferation after 24 h treatment. 1,25-D3 at various concentrations treatment promoted cell proliferation in a dose-dependent manner (Figure 1(A)). In line with CCK-8 assay, LDH releasing assay result showed that 1,25-D3 inhibited *H. pylori*-induced LDH releasing in GES-1 cells (Figure 1(B)).

### 1,25-D3 inhibits *H. pylori-*induced cell apoptosis in GES-1cells

**Figure 2.**
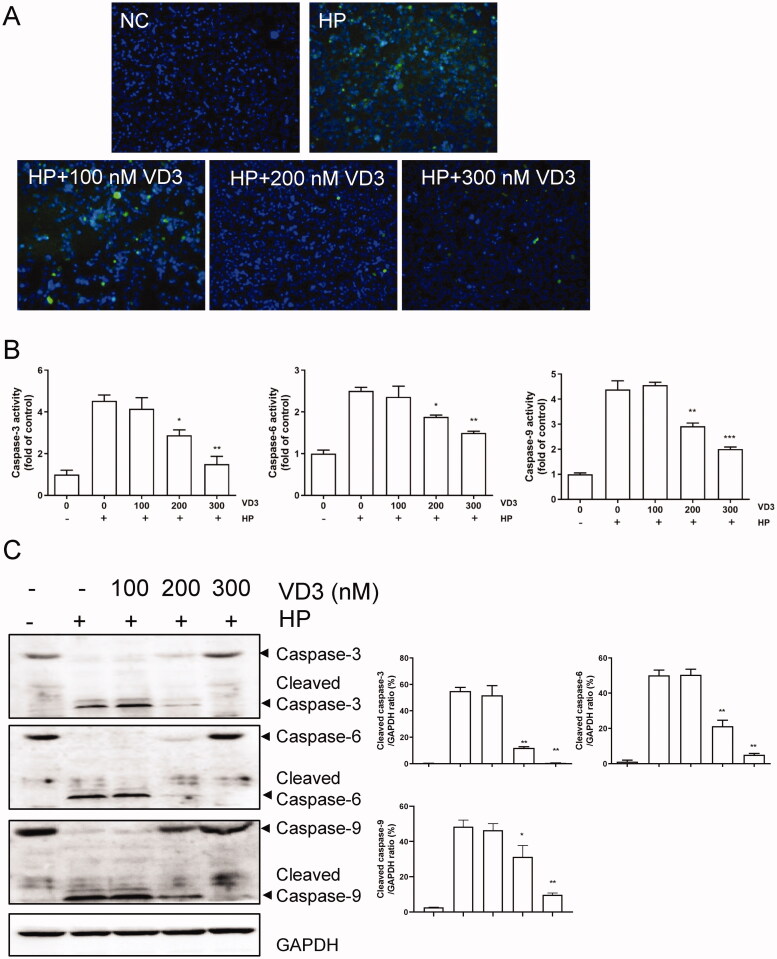
1,25-D3 inhibits *H. pylori*-induced cell apoptosis in GES-1 cells. (A) GES-1 cells were infected with *H. pylori* SS1 strain (MOI: 100) and treated with different concentrations of 1,25-D3 for 24 h, the levels of apoptosis were analysed using an TUNEL detection kit. (B) GES-1 cells were infected with *H. pylori* SS1 strain (MOI: 100) and treated with different concentrations of 1,25-D3 for 24 h, caspase-3, caspase-6 and caspase-9 activities were determined by commercially available kits. (C) GES-1 cells were infected with *H. pylori* SS1 strain (MOI: 100) and treated with different concentrations of 1,25-D3 for 24 h, caspase-3, caspase-6 and caspase-9 expression were determined by western blot. Bars represent means ± S.E.M of three independent experiments. *p < 0.01 vs. *H. pylori* treatment.

Next, we detected the effect of 1,25-D3 on *H. pylori*-induced cell apoptosis in GES-1cells. Consistent with CCK-8 and LDH releasing results, TUNEL assay result showed that *H. pylori* caused a significant apoptosis in GES-1 cells after 24 h treatment. 1,25-D3 prevented *H. pylori*-induced cell apoptosis in GES-1 cells with a less positive cells (Figure 2(A)). Caspase activity assay results showed that 1,25-D3 inhibited *H. pylori*-induced caspase-3, caspase-6 and caspase-9 activities in GES-1 cells (Figure 2(B)). In addition, the levels of cleaved caspase-3, −6 and −9 were determined by western blot. the results showed that 1,25-D3 significantly inhibited *H. pylori*-induced cleaved caspase-3, −6, −9 expression in GES-1 cells (Figure 2(C)).

### Mitochondrial pathway is involved in the anti-apoptotic effect of 1,25-D3 in *H. pylori-*infected GES-1 cells

**Figure 3.**
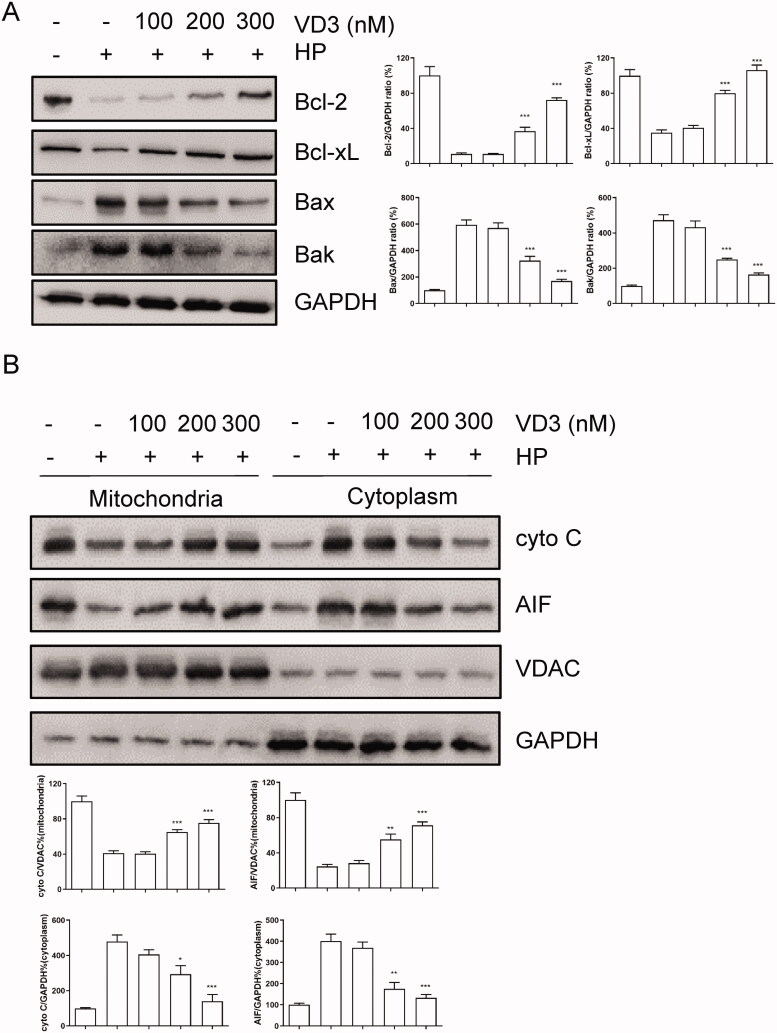
Bcl-2 families are involved in the anti-apoptotic effect of 1,25-D3 in *H. pylori*-treated GES-1cells. (A) GES-1 cells were infected with *H. pylori* SS1 strain (MOI: 100) and treated with different concentrations of 1,25-D3 for 24 h, Bcl-2, Bcl-xL, Bax and Bak levels were determined by western blot. (B) GES-1 cells were infected with *H. pylori* SS1 strain (MOI: 100) and treated with different concentrations of 1,25-D3 for 24 h, Cytochrome C (Cyto C) and apoptosis inducing factor (AIF) levels were determined by western blot. Bars represent means ± S.E.M of three independent experiments. *p < 0.01, **p < 0.05, ***p < 0.001 vs. *H. pylori* alone treatment.

Furthermore, we aimed to confirm the effect of 1,25-D3 on apoptosis in *H. pylori*-infected GES-1 cells. Anti- or pro-apoptotic protein levels were determined by western blot assay. As shown in Figure 3(A), after 24-h treatment, *H. pylori* caused Bcl-2 and Bcl-xL expression levels significantly decreasing in GES-1 cells. Meanwhile, *H. pylori* caused Bax and Bak expression levels significantly increasing, indicating that *H. pylori* caused GES-1 cells apoptosis through a mitochondrial-dependent pathway. 1,25-D3 treatment prevent against *H. pylori*-induced apoptosis through regulation of Bcl-2 families expression (Figure 3(A)). We attempted to isolate the nuclear and cytoplasm and detected the levels of Cyto C and AIF, which are related to mitochondrial pathway. The results showed that 1,25-D3 prevented Cyto C releasing from mitochondria (Figure 3(B)).

### 1,25-D3 promotes c-Raf/MEK/ERK phosphorylation in *H. pylori-* infected GES-1 cells

**Figure 4.**
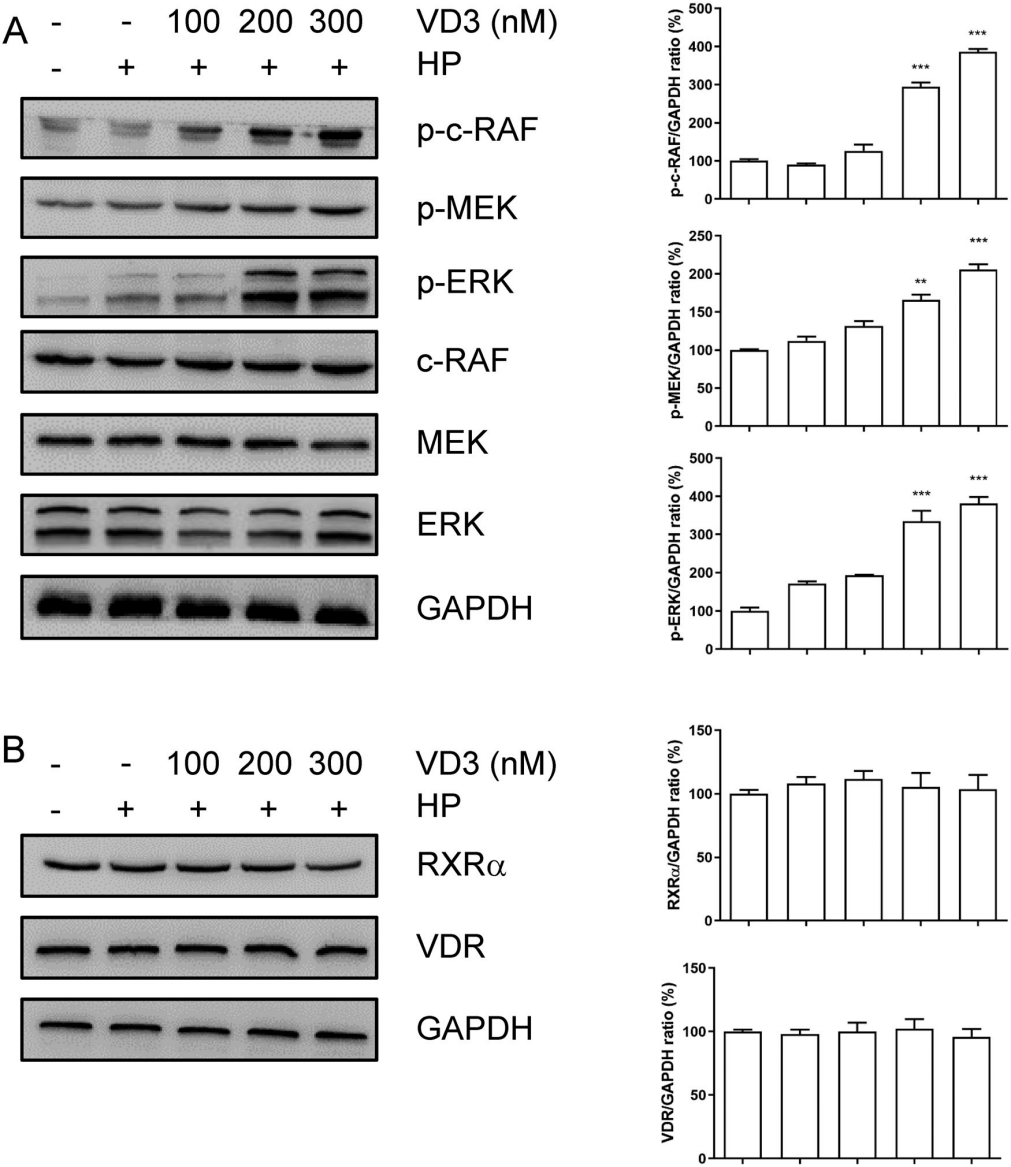
1,25-D3 promotes c-Raf/MEK/ERK phosphorylation in *H. pylori*-treated GES-1 cells. GES-1 cells were infected with *H. pylori* SS1 strain (MOI: 100) and treated with different concentrations of 1,25-D3 for 24 h, (A) c-Raf, MEK and ERK phosphorylation levels and (B) RXRα and VDR levels were determined by western blot. Bars represent means ± S.E.M of three independent experiments. **p < 0.01, ***p < 0.001 vs. *H. pylori* alone treatment.

A previous study has shown that VD3 promotes intercellular c-Raf/MEK/ERK phosphorylation. As shown in Figure 4(A), 1,25-D3 promoted c-Raf, MEK and ERK phosphorylation in *H. pylori*-infected GES-1 cells with a dose-dependent manner. Moreover, 1,25-D3 did not change the levels of total c-Raf, MEK and ERK in GES-1 cells. VDR as a transcriptional factor regulated gene expression with RXRα. However, 1,25-D3 did not change the levels of RXRα and VDR in GES-1 cells (Figure 4(B)).

### 1,25-D3 exerts an anti-apoptotic effect in *H. pylori-*treated GES-1 cells through binding to VDR

**Figure 5.**
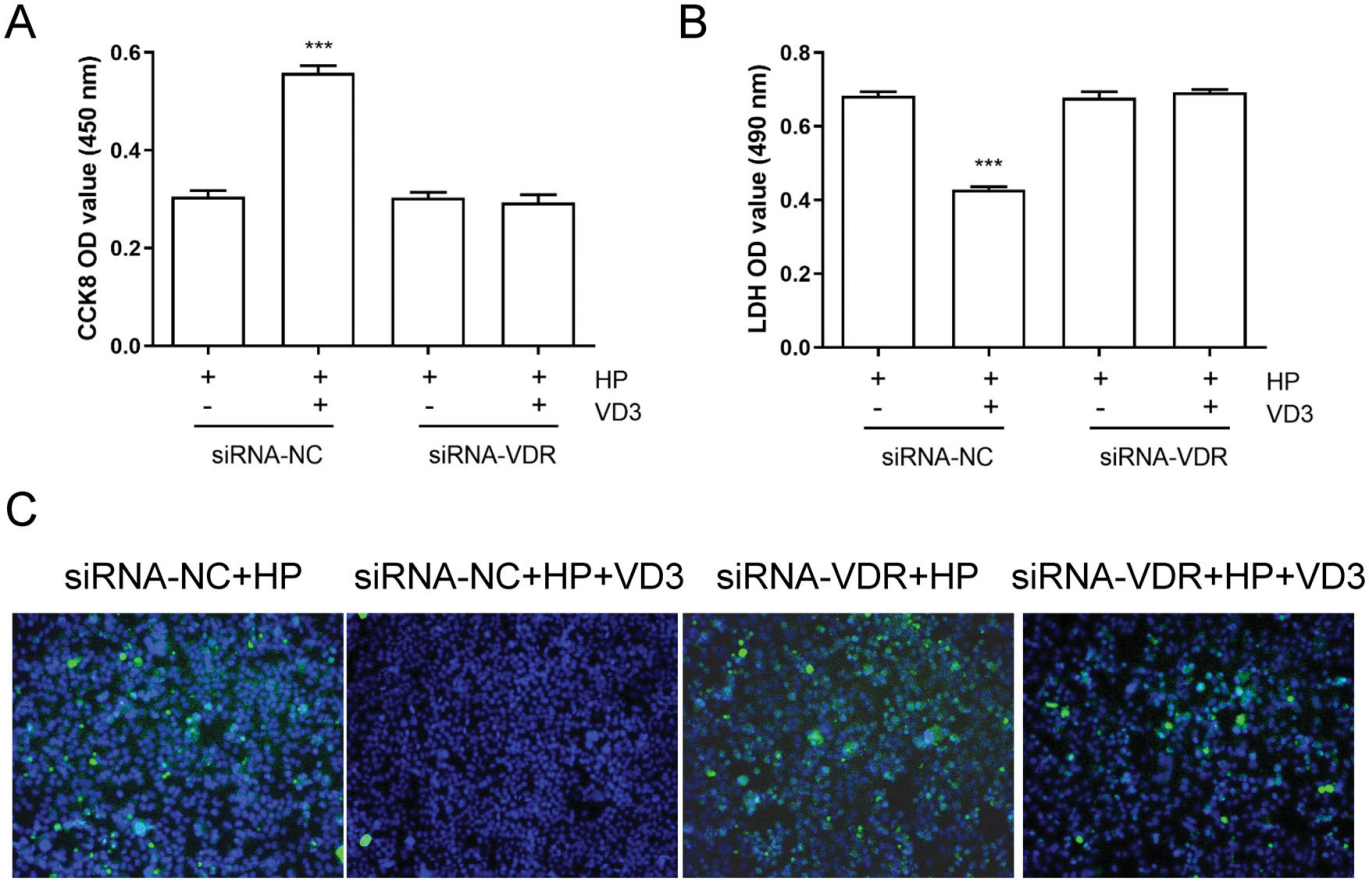
1,25-D3 exerts an anti-apoptotic effect in *H. pylori*-treated GES-1 cells through binding to VDR. GES-1 cells were transfected with siRNA-VDR for 24 h, and then infected with *H. pylori* SS1 strain (MOI: 100) and treated with different concentrations of 1,25-D3 for 24 h, (A) the levels of VDR expression was determined by western blot, (B) the cell viability was determined by CCK-8 assay, (C) LDH release was determined by a commercial kit and (D) the levels of apoptosis were analysed using an TUNEL detection kit. Bars represent means ± S.E.M of three independent experiments. ***p < 0.001 vs. *H. pylori* treatment + siRNA-NC.

VDR activated by 1,25-D3 plays critical roles in many physiological functions. In order to determine the effect of VDR on the anti-apoptotic effect of 1, 25D3, we used siRNA to knockdown the expression of VDR in GES-1 cells. As shown in Figure 5, knockdown of VDR did not affect the cell viability, LDH releasing and apoptosis of *H. pylori*-infected GES-1 cells. However, 1,25-D3 treatment caused significant changes in cell viability, LDH releasing and apoptosis levels in siRNA-VDR cells compared with siRNA-NC cells, indicating that 1,25-D3 exerts an anti-apoptotic effect in *H. pylori*-treated GES-1 cells through binding to VDR.

### The inhibition of c-Raf/MEK/ERK phosphorylation blocks the anti-apoptotic effect of 1,25-D3 in *H. pylori-*treated GES-1 cells

**Figure 6.**
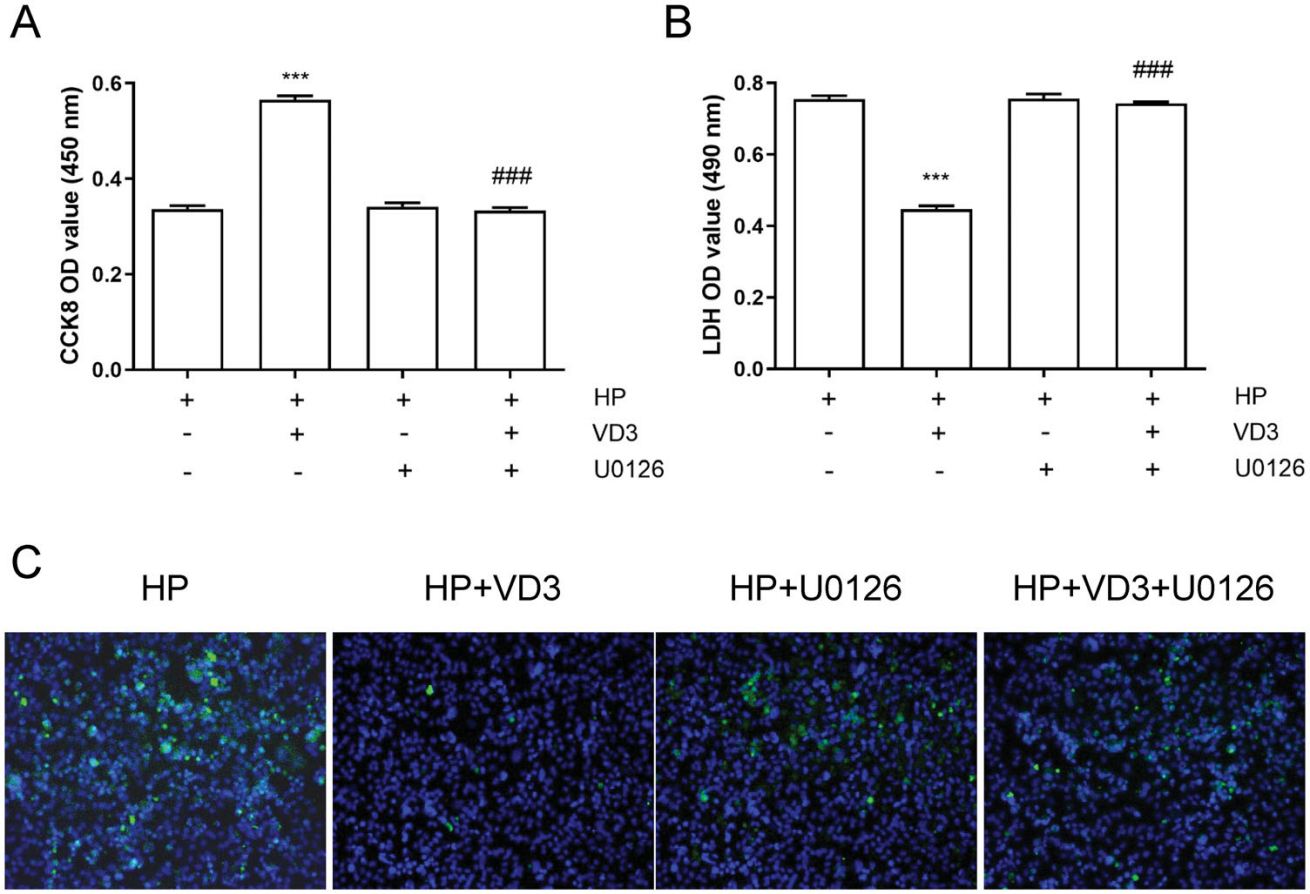
The inhibition of c-Raf/MEK/ERK phosphorylation blocks the anti-apoptotic effect of 1,25-D3 in *H. pylori*-treated GES-1 cells. GES-1 cells were treated with MEK inhibitor (U0126) for 24 h, and then infected with *H. pylori* SS1 strain (MOI: 100) and treated with different concentrations of 1,25-D3 for 24 h, (A) the cell viability was determined by CCK-8 assay, (B) LDH release was determined by a commercial kit and (C) the levels of apoptosis were analysed using an TUNEL detection kit. Bars represent means ± S.E.M of three independent experiments. ***p < 0.001 vs. *H. pylori* alone treatment, ###*p* < 0.001 vs. *H. pylori* + 1,25-D3 treatment.

Previous results showed that 1,25-D3 promotes c-Raf/MEK/ERK phosphorylation in *H. pylori*-infected GES-1 cells. Hence, we used MEK inhibitor U0126 to block c-Raf/MEK/ERK pathway phosphorylation pharmacologically. As shown in Figure 6, U0126 alone treatment did not alter cell viability, LDH releasing and apoptosis. 1,25-D3 failed to promote cell viability, decrease LDH release and apoptosis in U0126 treated cells. The results indicated that 1,25-D3 exerts an anti-apoptotic effect in *H. pylori*-treated GES-1 cells through promoting c-Raf/MEK/ERK phosphorylation.

### 1,25-D3 protects against *H. pylori-*infected apoptosis through a vitamin D receptor-dependent c-Raf/MEK/ERK pathway in mice

**Figure 7.**
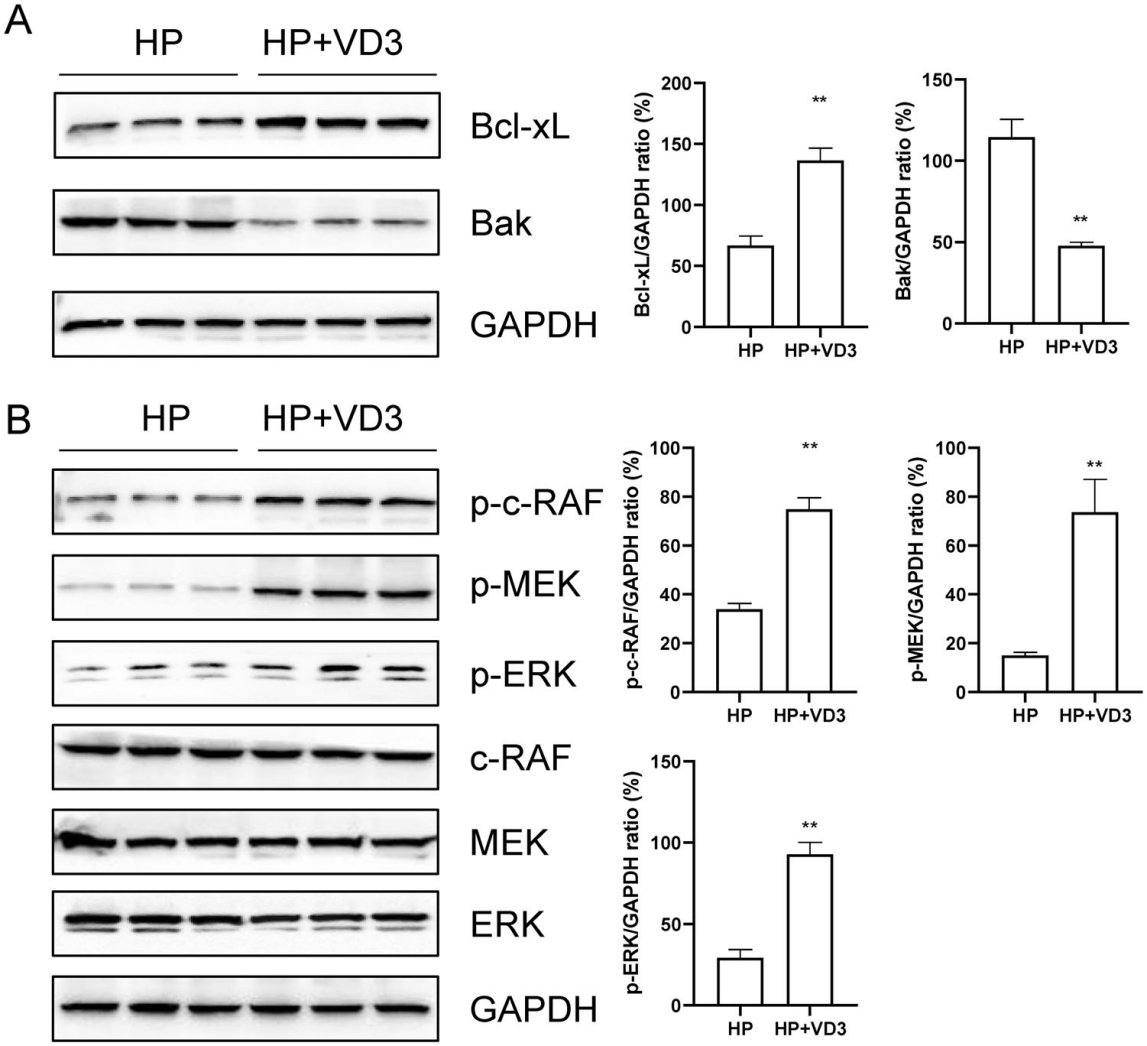
1,25-D3 protects against *H. pylori*-infected apoptosis through a vitamin D receptor-dependent c-Raf/MEK/ERK pathway in mice. Mice were orally gavaged with 10^8^ CFUs of *H. pylori* and 25 µg/kg 1,25-D3 every other day for 1 month. Bcl-xL, Bak, c-Raf, MEK and ERK phosphorylation levels in the stomach of mice were determined by western blot. Bars represent means ± S.E.M of three random mice. **p < 0.01 vs. *H. pylori* alone treatment.

To determine the effects of 1,25-D3 on *H. pylori*-infected apoptosis, mice were orally gavaged with 10^8^ CFUs of H. pylori or 25 µg/kg 1,25-D3 every other day for 1 month. The tissues from stomach were harvested and the protein expression levels of Bcl-xL, Bak, p-c-RAF, p-MEK, p-ERK, c-RAF, MEK and ERK were determined by western blots. The results showed that 1,25-D3 treatment significantly inhibited Bak protein expression and increased Bcl-xL, p-c-RAF, p-MEK and p-ERK protein expression (Figure 7). These results indicated that, *in vivo*, 1,25-D3 protected against *H.pylori*-infected apoptosis through a c-Raf/MEK/ERK pathway in mice.

## Discussion

In this study, we demonstrated that the role of 1,25-D3, a biologically active form of VD3, in *H. pylori*-infected gastric mucosa epithelial cells. Results showed that 1,25-D3 promoted cell proliferation and reduced LDH releasing in *H. pylori*-infected GES-1 cells. Moreover, 1,25-D3 inhibited *H. pylori*-induced cell apoptosis in GES-1 cells through regulation of Bcl-2 families expression as well as the inhibition of caspase activities. Importantly, 1,25-D3 promotes c-Raf/MEK/ERK phosphorylation in *H. pylori*-infected GES-1 cells. RNA silencing results showed that suppression of 1,25-D3 in apoptosis was relying on binding to VDR. Blocking of c-Raf/MEK/ERK phosphorylation by MEK inhibitor abolished the anti-apoptotic effect of 1,25-D3. Herein, our results demonstrated that 1,25-D3 protected gastric mucosa epithelial cells against *H. pylori*-induced apoptosis through a VDR-dependent c-Raf/MEK/ERK pathway. Our findings may help to eradicate *H. pylori* following the standard quadruple therapy.

It is well known that antibiotic therapy leads to imbalance of intestinal microflora in patients and mild or severe episodes of antibiotic-associated diarrhoea (Bergogne-Bérézin 2000). Therefore, it is important to find the new option to eradicate this pathogenic bacterium from the host. For the gastric epithelium, it is crucial and difficult to maintain the integrity of the mucosa lining of the human gastrointestinal tract after *H. pylori* infection (Cover and Blaser 1992). The unique feature of *H. pylori* is due to the presence of urease, which is a special enzyme to catalyse the hydrolysis of urea to ammonia and forming the alkaline microenvironment, counterbalancing the deleterious effect of gastric acid on the bacteria (Crabtree et al. 1991; Dunn et al. 1997). A range of stimulis such as hormones, cytokines and exogenous origin are involved in the reaction of the epithelial cells and gastric mucosal epithelium is the front line defence against *H. pylori* infection (Westblom 1997). To protect gastric mucosal epithelium from *H. pylori* triggered chronic gastritis, peptic ulcers blocks the development of mucosa-associated lymphoid tumours and gastric adenocarcinoma (Lee et al. 2016). Apoptosis, which is characterised by cell shrinkage, membrane blebbing, nuclear breakdown, and DNA fragmentation, is indispensable for tissue homeostasis and activation of the immune system (Moss 1998). In this study, we found that 1,25-D3 prevented *H. pylori*-induced cell proliferation decreases, and even reduced apoptosis rate in *H. pylori*-infected gastric mucosa epithelial cells through mitochondrial-dependent pathway. To date, few studies have reported that 1,25-D3 has protective effect against apoptosis through mitochondrial pathway. A previous study showed that 1,25-D3 induced apoptosis in breast cancer cells and tumours in a caspase-independent pathway (Westblom 1997).

Interestingly, we found 1,25-D3 prevented caspase activities increasing against apoptosis. It has been reported that *H. pylori* induces increased expression of the VDR and CAMP in immune responses and reduced cytokine activation in GES-1 cells infected with *H. pylori* (Guo et al. 2014). However, in our study, VDR expression in gastric mucosa epithelial cells did not alter both *H. pylori* infection and 1,25-D3 treatment as well as RXRα expression.

## Conclusions

Numerous studies have reported that the VD/VDR signalling pathway play a key role in many physiological process (Christakos et al. 2016). VD as a VDR ligand binds to VDR to cause c-Raf/MEK/ERK phosphorylation (Han et al. 2010). It was found that 1,25-D3 promoted c-Raf/MEK/ERK phosphorylation in *H. pylori*-infected GES-1 cells. *H. pylori* activates matrix metalloproteinase 10 in gastric epithelial cells via EGFR and ERK-mediated Pathways (Costa et al. 2016). It was found that 1,25-D3 enlarged the phosphorylation level of c-Raf, MEK and ERK in *H. pylori*-infected GES-1 cells, which may contribute to cell proliferation and apoptosis. Physiological process of cell death, apoptosis, plays a beneficial role in organism survival (Pierzchalski et al. 2009). However, in some pathologies, like gastric *H. pylori* infection, this process may turn against the host organism causing tissue damage. To find drugs controlling apoptosis may have potential significance in treatment of *H. pylori* infection. Overall, 1,25-D3 was identified to be effective and anti-apoptotic in infection caused by *H. pylori*. Mitochondrial-dependent pathway was involved in anti-apoptotic effect of 1,25-D3, and molecular mechanism of its was directed to the VDR-dependent c-Raf/MEK/ERK pathways. In summary, our findings defined a potential use of 1,25-D3 in *H. pylori* infection.
